# Type of organic fertilizer rather than organic amendment per se increases abundance of soil biota

**DOI:** 10.7717/peerj.11204

**Published:** 2021-05-07

**Authors:** Maria Viketoft, Laura G.A. Riggi, Riccardo Bommarco, Sara Hallin, Astrid R. Taylor

**Affiliations:** 1 Department of Ecology, Swedish University of Agricultural Sciences, Uppsala, Sweden; 2 Department of Forest Mycology and Plant Pathology, Swedish University of Agricultural Sciences, Uppsala, Sweden

**Keywords:** Earthworms, Farmyard manure, Hay, Long-term field experiment, Microarthropods, Microorganisms, Nematodes, Sewage sludge, Soil food-web, Household compost

## Abstract

Addition of organic amendments is a commonly used practice to offset potential loss of soil organic matter from agricultural soils. The aim of the present study was to examine how long-term addition of organic matter affects the abundance of different soil biota across trophic levels and the role that the quality of the organic amendments plays. Here we used a 17-year-old fertilization experiment to investigate soil biota responses to four different organic fertilizers, compared with two mineral nitrogen fertilizers and no fertilization, where the organic fertilizers had similar carbon content but varied in their carbon to nitrogen ratios. We collected soil samples and measured a wide range of organisms belonging to different functional groups and trophic levels of the soil food web. Long-term addition of organic and mineral fertilizers had beneficial effects on the abundances of most soil organisms compared with unfertilized soil, but the responses differed between soil biota. The organic fertilizers generally enhanced bacteria and earthworms. Fungi and nematodes responded positively to certain mineral and organic fertilizers, indicating that multiple factors influenced by the fertilization may affect these heterogeneous groups. Springtails and mites were less affected by fertilization than the other groups, as they were present at relatively high abundances even in the unfertilized treatment. However, soil pH had a great influence on springtail abundance. In summary, the specific fertilizer was more important in determining the numerical and compositional responses of soil biota than whether it was mineral or organic. Overall, biennial organic amendments emerge as insufficient, by themselves, to promote soil organisms in the long run, and would need to be added annually or combined with other practices affecting soil quality, such as no or reduced tillage and other crop rotations, to have a beneficial effect.

## Introduction

Soil biodiversity is highlighted in the European soil thematic strategy as a key component of soil quality (European Commission, 2002), because soil organisms provide key ecosystem functions, such as decomposition, nutrient and carbon (C) cycling and water regulation (Barrios, 2007; Kulmatiski et al., 2014). Worldwide, the quality of agricultural soils is threatened by the loss of soil organic matter (SOM) and by soil compaction (Montanarella, 2015). A practice commonly used that offsets these threats and enhances soil functions is the application of carbon-containing amendments, i.e., organic fertilizers. A first step towards understanding how fertilization regulates soil quality is to examine its effects on the soil biota (Bender, Wagg & Van der Heijden, 2016). Organic fertilizers increase SOM and have positive immediate within season effects on the abundance of several soil organisms at different trophic levels, e.g., microorganisms (Birkhofer et al., 2008; Paterson et al., 2011), soil fauna such as nematodes (Ferris & Matute, 2003; Paterson et al., 2011), mites (Axelsen & Kristensen, 2000; Cao et al., 2011), collembolans (Axelsen & Kristensen, 2000; Haubert et al., 2009) and earthworms (Lofs-Holmin, 1983), as well as aboveground predators (Hines, Megonigal & Denno, 2006; Riggi & Bommarco, 2019). On the other hand, mineral nitrogen (N) fertilization has been reported to have minimal immediate impact on soil microbes (Geisseler & Scow, 2014) and inconsistent effects on soil fauna e.g., microarthropods (Siepel & Van de Bund, 1988; Curry, 1994; Postma-Blaauw et al., 2010). However, while the general within season response to organic fertilization is enhanced diversity and abundance of soil biota when compared with no or mineral fertilization, it is less well known how the overall soil communities react to long-term organic fertilization.

In contrast to the documented positive immediate effects of organic amendments on soil organisms, their long-term effects are inconsistent. For example, the biomass of microbial primary decomposers (Wander et al., 1995; Marschner, Kandeler & Marschner, 2003; Bünemann, Schwenke & Van Zwieten, 2006) and the abundances of secondary decomposers (Andrén & Lagerlöf, 1983; Moore, 1994; Alvarez, Frampton & Goulson, 2001; Kautz, López-Fando & Ellmer, 2006; Ngosong et al., 2009) can be both positively and negatively affected by long-term organic fertilization. These incongruences could be due to differences in the quality of the applied organic matter. In particular, labile carbon sources with a low C/N ratio constitute a high-quality C source for the lower trophic levels in the soil food web (Bardgett et al., 2005; Haynes, 2005; Cotrufo et al., 2013; de Vries & Caruso, 2016). Organic fertilizers that increase labile C inputs are therefore used more efficiently by microbes than recalcitrant fertilizers, and in the long term, high-quality labile litter results in greater SOM formation and accumulation (Cotrufo et al., 2013). Nevertheless, to our knowledge comparative investigations of organic fertilizers with respect to their qualitative effects of available C (i.e., equal amounts of C) on soil organisms are scarce (Leroy, 2008), and even lacking for long-term conditions.

To assess whether organic fertilizers are better at enhancing soil biota than mineral fertilizers in the long-term, we used a 17-year-old fertilization experiment in which all management practices other than type of fertilizer were similar. The amount of added C was comparable across all organic fertilizers, but the quality (i.e., C/N) of the organic amendment varied depending on type of organic matter added (manure, hay, compost or sewage sludge). To primarily assess the long-term effects of the organic amendments, soil samples were collected 1.5 years after the last application of organic amendments. We expected (1) mineral fertilizers to have an indirect positive effect on the abundance of soil biota as a consequence of increased crop growth from fertilization (Kätterer, Börjesson & Kirchmann, 2014), and (2) organic fertilizers to have an even greater positive effect on the abundance of soil biota as they provide a direct source of C in addition to the indirect C input via crop growth (Bünemann, Schwenke & Van Zwieten, 2006). We also anticipated (3) the organic fertilizers to differ in their effect due to differing N concentrations, i.e., C/N ratios. Microorganisms, in particular bacteria, likely respond positively to the amendments with lower C/N ratio (Leite et al., 2017), i.e., sewage sludge (C/N = 8.8) and compost (C/N = 9.4). On the other hand, earthworms should respond positively to the amendments with higher C/N (Aira, Monroy & Domínguez, 2006), i.e., hay (C/N = 20.5) and manure (C/N = 16.8), whereas nematodes, springtails and mites were expected to show varying responses due to differing food sources within each of these faunal groups.

## Materials & Methods

### Experimental field site

In 2014, we conducted our study at a long-term agricultural fertilization experiment established in 1996 at the Lanna agricultural research station (58.34° N, 13.10° E) in Western Sweden. The experiment consists of nine treatments in a randomized block design with four blocks (i.e.,  *n* = 4), and each individual plot area comprises 112 m^2^. We sampled seven of these treatments (7 × 4, giving a total of 28 plots): four treatments with organic matter fertilization (compost, manure, hay, and sewage sludge), two mineral nitrogen (N) fertilizer treatments (calcium nitrate (Ca(NO_3_)_2_) and ammonium sulphate ((NH_4_)_2_SO_4_)), and one unfertilized treatment. All organic fertilizer treatments received the same application rate of 8 Mg ash-free dry matter ha^−1^, so that similar amounts of C were added across all organic fertilizers. However, the quality (i.e., C/N) varied due to the type of organic matter added (Table 1, Table S1). From 1996 onwards, the organic amendments were applied every second year in autumn after harvest and before ploughing. This means the last application prior to the sampling was done in the autumn of 2012. Every spring, the N-fertilized plots received 80 kg N ha^−1^. In addition, these plots also received 20 kg P ha^−1^ and 15 kg K ha^−1^ in spring 1995 and 1996, but from 1998 onwards the P and K were applied biannually in autumn with 40 kg P and 30 kg K ha^−1^.

**Table 1:** Fertilizer treatments in the Lanna long-term field experiment, C/N of the organic amendments and measured soil chemical factors. Mean soil pH, total carbon (Tot C), total nitrogen (Tot N) and C/N of the soil in 2014 (https://www.slu.se/institutioner/mark-miljo/forskning/vaxtnaringslara-/vaxtnaring/langliggan de-vaxtnaringsforsok/r3-0130/). Different letters within each soil factor (column) indicate significant treatment effects (lme, Tukey *p* < 0.05, *n* = 4).

**Treatment**	**C/N** **amendment**	**pH** **(H**_**2**_**O)**	**Tot C** **(%)**	**Tot N** **(%)**	**C/N** **soil**
Unfertilized	–	6.24 b	2.01 e	0.17 d	11.7 a
Calcium nitrate (Ca(NO_3_)_2_)^1^	–	6.37 ab	2.07 de	0.17 d	11.9 a
Ammonium sulphate ((NH_4_)_2_SO_4_)^1^	–	5.89 c	2.09 cd	0.18 cd	11.7 a
Grass hay^2^	20.5	6.23 b	2.13 c	0.18 c	11.7 a
Farmyard manure^3^	16.8	6.31 ab	2.41 b	0.20 b	11.8 a
Household compost^4^	9.4	6.49 a	2.70 a	0.25 a	10.9 b
Sewage sludge^5^	8.8	5.98 c	2.35 b	0.21 b	11.4 a

**Notes.**

1 80 kg N ha ^−1^ yr ^−1^, but in 1996 only 40 kg N due to residual N after peas grown in 1995.

2 Consisted mainly of Timothy.

3 From a cowshed with straw bedding.

4 Derived from domestic waste.

5 Dewatered anaerobically digested sewage sludge, only low levels of toxicants in the sludge (Börjesson, Kirchmann & Kätterer, 2014).

During the period 1997–2014, the mean annual temperature at the site was 9.1 °C and the mean annual precipitation was 455 mm. The soil developed on a Quaternary silty clay deposit with a clay content of 42% (Börjesson, Kirchmann & Kätterer, 2014). At the start of the experiment, the topsoil (0–20 cm) had an average pH of 6.6, approx. 2% C and a C/N of 11.1 (Kätterer, Börjesson & Kirchmann, 2014). The chemical properties of the soil have been affected by the fertilization treatments, with addition of sewage sludge and ammonium sulphate causing a decrease in pH and addition of compost causing an increase in total carbon and nitrogen (Table 1). In addition, the physical properties of the soil differ between the fertilization treatments; for example bulk density is lowest in sewage sludge (1.30 g cm^−3^) and highest in the unfertilized control (1.38 g cm^−3^) (Kätterer, Börjesson & Kirchmann, 2014). Since the start of the experiment, only cereals have been cultivated: oats (6 years), spring barley (7 years) and winter wheat (4 years), and in 2014 the crop was winter wheat. Aboveground biomass is removed after each harvest followed by moldboard ploughing in the autumn. The fertilizer treatments have also affected the grain yield, with sewage sludge and mineral fertilizers giving the highest average dry matter grain yield and the unfertilized control the lowest (Kätterer, Börjesson & Kirchmann, 2014). This difference in yield also has affected the mean annual C input to the soil, with C input increasing from no fertilization <mineral fertilizers <hay <compost <manure <sewage sludge (Kätterer, Börjesson & Kirchmann, 2014).

Soil organisms were sampled as long as possible after the application of the organic amendments to minimize their short-term, immediate effects and to primarily assess the long-term effects. Therefore, all soil and fauna samples were collected during the growing season of 2014, i.e., more than 1.5 years after the last organic amendment in the autumn of 2012.

### Microbial abundances

Soil samples for the estimation of microbial abundances were collected in mid-June 2014. Per plot, five samples were taken with a soil corer (diam. 2 cm, 10 cm depth) and combined into a composite sample. DNA was extracted from a total of 0.40 g fresh soil, using a FastDNA SPIN kit for soil according to the manufacturer’s instructions (MP Biomedicals, Santa Ana, CA USA). The quality and size of the DNA was checked by agarose–gel electrophoresis and quantified using a Qubit^®^ fluorometer and Qubit^®^ dsDNA BR assay kit (Life Technologies Corporation, Carlsbad, CA USA). Comparable amounts of DNA were extracted from each soil sample (35.7 ±5 SD ng mg^−1^ DW of soil, *n* = 36, min = 21.4 and max = 44.7).

The abundances of bacterial and fungal communities in each plot were determined by quantitative real-time PCR (qPCR) using domain-specific 16S rRNA and ITS primers previously described for bacteria (López-Gutiérrez et al., 2004) and fungi (Ihrmark et al., 2012). For each sample, two independent qPCR reactions in two different runs were performed using the BioRad CFX Connect Real-Time System (BioRad, Hercules CA, USA). The total reaction volume was 15 µL, including 2X iQ™ SYBR Green supermix (BioRad), 0.1% bovine serum albumin (New England Biolabs, Ipswich, MA, USA), primers (0.5 µM of each primer for 16S rRNA and 0.3 µM for ITS) and 10 ng soil DNA. Fluorescent signals were acquired at 78 °C and 79 °C for the 16S rRNA and ITS, respectively.

Standard curves for each assay were generated by serial dilutions of linearized plasmids with cloned fragments of 16S rRNA genes from *Pseudomonas aeruginosa* PAO1 and an ITS fragment cloned from an environmental sample (roots of *Rosa rugosa*). Standard curves were linear (*R*^2^ = 0.99) in the range used. The amplifications were verified by melting curve analysis and agarose gel electrophoresis, and non-template controls resulted in negligible values. Potential inhibition in all PCR reactions was checked by amplifying a known amount of the pGEM-T plasmid (Promega, Madison, WI, USA) with the plasmid specific T7 and SP6 primers when added to the DNA extracts or non-template controls. No inhibition was detected.

### Soil fauna sampling and extraction

Samples for microarthropods, i.e., springtails (Collembola) and mites (Acari), were collected at the end of April 2014 at four sampling points per plot using steel frames (10 × 10 cm, depth 10 cm). Microarthropods were extracted over a period of 72 h using funnel extractors (Tullgren, 1918), and were collected and stored in plastic jars filled with 70% ethanol. In each sample, adult collembolans and mites were identified to the sub-family and sub-order level, respectively, following Fjellberg (1998; 2007) and Krantz & Walter (2009), while juveniles were identified to sub-class. The abundance of collembolans and mites was expressed as individuals per m^2^, and a plot average was calculated based on the four samples.

Nematodes and earthworms were sampled in mid-September 2014. For nematodes, five soil cores (diam. 2 cm, depth 10 cm) were taken per plot and these were pooled, resulting in four samples per treatment. Samples were stored at 5 °C until extraction using a modified Baermann method (Viketoft et al., 2005). Nematodes were extracted from two 15 g fresh weight subsamples from the pooled sample. After an extraction time of 30 h, nematodes were heat-killed and preserved in 4% formaldehyde. Nematodes were counted and assigned to one of the following trophic groups: root feeders, bacterivores, fungivores, omnivores and predators. Abundances of nematodes were calculated as number of individuals per gram dry soil.

To estimate the earthworm populations, one soil sample (30 × 30 cm) per plot was taken down to a depth of 25 cm and hand sorted in the field. In addition, an AITC solution was applied at the bottom of the excavated hole to extract anecic earthworms (Pelosi et al., 2009). All earthworms were counted and determined to genus-level, and the total earthworm abundance was calculated as individuals per m^2^.

### Biomass determination

Besides determination of abundance, body masses were also estimated for springtails, mites and earthworms as intensive management practices may select for soil arthropod communities with particular life history traits (Birkhofer et al., 2017), such as small body size, and may decrease earthworm biomass (Singh et al., 2021). The body length of each springtail was measured under a microscope and, for each sample (four samples per plot), the mean body length of each collembolan sub-family was estimated. These means were used to calculate the body dry weight using the length/weight regression equations of Rosswall, Persson & Lohm (1977) and Persson et al. (1980). For mites, the dry weight was derived from dry weight data collected by Rosswall, Persson & Lohm (1977) and Persson et al. (1980). The community weighted means of body mass (CWB) were calculated as the ratio of the total dry weight and the total abundance in each sample of mites and collembolans, respectively (Tsiafouli et al., 2015). The biomass of earthworms was determined by weighing after drying at 105 °C for 24 h.

### Statistical analyses

We analyzed the effect of the fertilizer treatments on the abundance of each organism group and the biomass of springtails, mites and earthworms with linear mixed effects models. To compare the two fertilizer regimes specifically, we also performed a similar analysis but with only organic and mineral fertilizers included. The statistical models included random effects of block, to account for position within the field, and of plot, for cases with multiple samples within plots. Post-hoc differences between treatment means were examined with Tukey’s test (*p* < 0.05). Log values were used for the analyses of bacterial and fungal abundances. For soil faunal groups, data normality and homoscedasticity were checked visually using qqplots and residuals plots prior to analysis and a logarithmic transformation was used to homogenize variances when necessary. Analyses were performed with the procedure lme from the package lme4 in R version 3.1.1 (R Core Team, 2014). Correlations between C/N of the organic amendments and soil organisms were analysed using Spearman rank order correlation coefficients. The relationship between all abundances of the soil organism groups or biomass of a subset of the groups and all soil chemical factors were examined with Redundancy Analysis (RDA) in CANOCO 5. The control plots were included in the RDA, the analyses were centered and standardized by taxa and the results were tested on the basis of 499 unrestricted permutations. For the soil organism groups where several samples had been collected per plot, the average value of the samples was used in these analyses.

## Results

### Microorganisms

**Figure 1:**
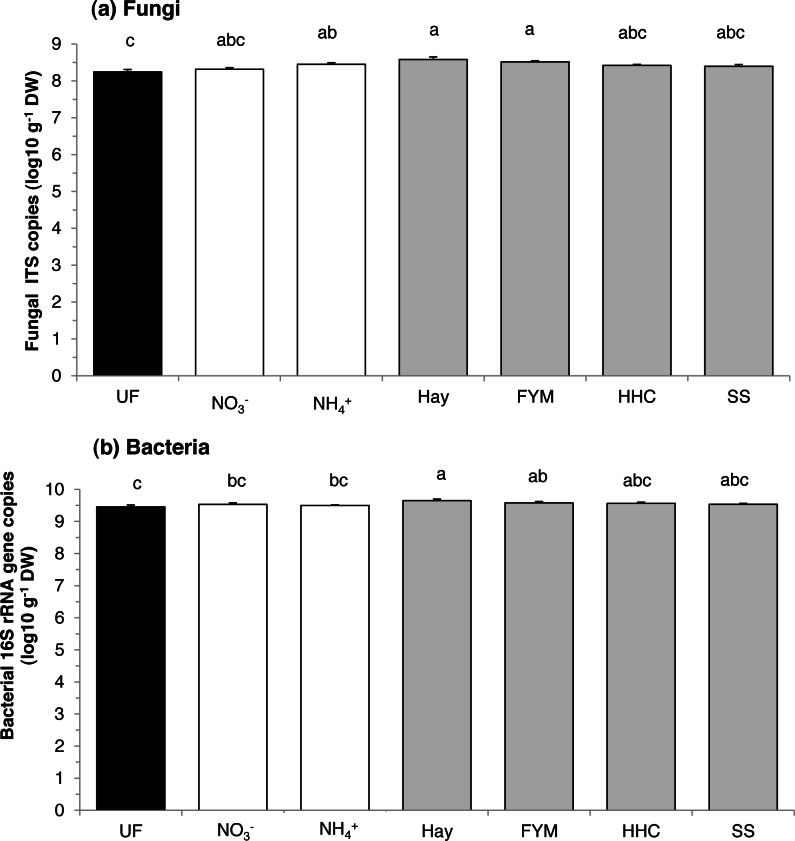
Mean abundances of (a) fungi and (b) bacteria in a long-term field experiment with different fertilizer treatments. Unfertilized (black column), mineral fertilizers (white columns) and organic fertilizers (grey columns). Treatment abbreviations: UN, unfertilized; NO${}_{3}^{-}$, mineral fertilizer Ca(NO_3_)_2_; NH${}_{4}^{+}$, mineral fertilizer (NH_4_)_2_SO_4_; Hay, grass hay; FYM, farmyard manure; HHC, household compost; SS, sewage sludge. Columns with the same letter are not significantly different at level *p* ≤ 0.05. Error bars indicate SE, *n* = 4.

There was an effect of fertilization on abundance of both fungi and bacteria (*F* = 4.06, *p* = 0.010 and *F* = 3.60, *p* = 0.016, respectively). Compared with the unfertilized soil, fungi were similarly stimulated by both organic (hay and manure) and mineral fertilizers (ammonium sulphate) (Fig. 1A). However, fungal abundance in unfertilized plots was similar to that in calcium nitrate, compost and sewage sludge plots. On the other hand, bacteria were generally enhanced by organic fertilizers compared with mineral fertilizers (*F* = 4.64, *p* = 0.044), and especially by the addition of hay (Fig. 1B). However, bacterial abundance in unfertilized plots was similar to that in compost and sewage sludge plots. Overall, there was no effect of treatment on the fungi:bacteria ratio, which ranged between 0.04 and 0.14.

### Nematodes

**Figure 2:**
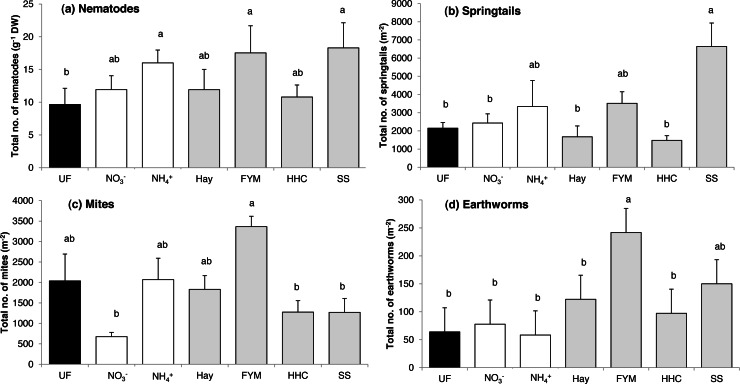
Mean abundances of (a) nematodes, (b) springtails, (c) mites and (d) earthworms in a long-term field experiment with different fertilizer treatments. Unfertilized (black column), mineral fertilizers (white columns) and organic fertilizers (grey columns). For treatment abbreviations, see Fig. 1. Columns with the same letter are not significantly different at level *p* ≤ 0.05. Error bars indicate SE, *n* = 4.

The total abundance of nematodes was affected by fertilization (*F* = 2.81, *p* = 0.041). Some mineral (ammonium sulphate) and some organic (sewage sludge, manure) fertilizers increased nematode abundance compared with unfertilized soil (Fig. 2A). Taken separately, none of the nematode feeding groups were affected by the fertilizer treatments (Table S2).

### Springtails and mites

The fertilizer treatments affected both total springtail (*F* = 4.70, *p* = 0.005) and total mite abundance (*F* = 4.43, *p* = 0.006). For springtails, there was no general difference in abundance between organic and mineral fertilizers, and in addition, in most treatments, abundances were similar to that in the unfertilized control (Fig. 2B). However, the sewage sludge treatment greatly increased the total abundance of springtails (Fig. 2B), which was mostly due to the Podumorpha that generally accounted for the majority of the springtail community (73%) (Table 2).

**Table 2:** Abundance of different groups of springtails and mites (individuals per m^2^) in a long-term fertilization experiment in Lanna; mean (SE). Different letters indicate significant treatment effects within each group (lme, Tukey *p* < 0.05, *n* = 4).

**Treatment**	**Springtails**			**Mites**			
	**Podumorpha**	**Entomo-** **bryomorph**a	**Symphy-** **pleona**	**Oribatida**	**Astigmata**	**Prostigmata**	**Mesostigmata**
Unfertilized	1544 (234) b	488 (124)	119 (45)	88 (29) ab	694 (458) a	181 (36) ab	113 (41) c
Ca(NO_3_)_2_	1788 (419) b	538 (118)	106 (30)	38 (19) b	25 (15) b	144 (36) b	194 (38) c
(NH_4_)_2_SO_4_	2263 (975) b	881 (440)	194 (50)	200 (68) a	181 (122) ab	150 (81) b	619 (79) ab
Grass hay	956 (376) b	613 (222)	106 (58)	19 (16) b	394 (136) a	175 (82) b	300 (20) bc
Farmyard manure	2406 (409) ab	1006 (292)	106 (18)	106 (34) ab	388 (100) a	506 (118) a	1413 (305) a
Household compost	1081 (317) b	263 (89)	131 (41)	19 (10) b	119 (60) ab	19 (16) b	694 (199) ab
Sewage sludge	5625 (1284) a	831 (99)	188 (56)	44 (22) b	175 (88) ab	175 (83) b	194 (57) c

The total mite abundance was generally enhanced by organic fertilizers compared with mineral fertilizers (*F* = 4.45, *p* = 0.038), although there were large variations between the treatments within each of the two fertilizer types (Fig. 2C). In addition, the unfertilized control supported an intermediate abundance, while the lowest mite abundance was found in the mineral nitrate treatment (Fig. 2C). At the subgroup level, Astigmata abundance was generally enhanced by organic fertilizers compared to mineral fertilizers (*F* = 8.62, *p* = 0.004), while Oribatida showed the opposite pattern with higher abundances in mineral fertilized plots than in organic fertilized plots (*F* = 6.37, *p* = 0.013). Significant differences between fertilized treatments and no-fertilization were confined for the Mesostigmata (Table 2). This mostly predatory mite group peaked in both mineral fertilization (ammonium sulphate) and organic fertilization (manure, compost) treatments, in which they accounted for the majority of the mite community (50–80%) (Table 2).

Total mesofauna biomass, i.e., the combined biomass of springtails and mites, was affected by the fertilizer treatments (*F* = 6.15, *p* = 0.0012), with a higher biomass in the manure fertilization compared with all other treatments except ammonium sulphate (Table 3). This is most probably a reflection of the high number of large-bodied mesostigmatic mites in this treatment (Table 2). There was no effect of fertilizer treatments on springtail CWB, while mite CWB differed between fertilizer treatments (*F* = 7.19, *p* = 0.0005) (Table 3) and generally mirrored the abundance patterns of the different mite groups. The lower mite CWB in the unfertilized treatment may be related to the mite community being dominated by astigmatic mites (Table 2), as this group often comprises smaller species. The large mite CWB in the compost, manure and the ammonium sulphate treatments was probably due to the dominance of large mesostigmatic mite species.

**Table 3:** Biomass of mesofauna (total and community weighted mean biomass (CWB) of springtails and mites) and earthworms in a long-term fertilization experiment in Lanna; mean (SE). Different letters indicate significant treatment effects within each group (lme, Tukey *p* < 0.05, *n* = 4).

**Treatment**	**Mesofauna** **(mg DW m**^−2^**)**	**Springtail CWB** **(µg/ind)**	**Mite CWB** **(µg/ind)**	**Earthworms** **(g DW m**^−2^**)**
Unfertilized	3.1 (0.6) b	0.6 (0.1)	0.9 (0.1) c	1.8 (0.7) b
Ca(NO_3_)_2_	3.6 (0.5) b	0.8 (0.1)	2.1 (0.1) abc	2.5 (0.9) b
(NH_4_)_2_SO_4_	7.4 (1.7) ab	0.7 (0.1)	3.3 (0.8) ab	2.1 (0.7) b
Grass hay	3.7 (0.7) b	0.5 (0.1)	1.8 (0.2) bc	1.8 (0.5) b
Farmyard manure	12.8 (1.8) a	0.6 (0.1)	3.3 (0.3) ab	5.9 (1.0) a
Household compost	5.8 (1.2) b	0.6 (0.1)	3.6 (0.4) a	1.7 (0.5) b
Sewage sludge	7.2 (1.9) b	0.7 (0.1)	1.4 (0.3) c	2.8 (0.8) ab

### Earthworms

In total, 249 earthworms belonging to the genera *Aporrectodea* and *Lumbricus* were found, with *Aporrectodea* clearly dominating (70–100%). The total abundance of earthworms was affected by the fertilizer treatments (*F* = 3.96, *p* = 0.011), and earthworms were enhanced by organic fertilizers compared with mineral fertilizers (*F* = 6.20, *p* = 0.022), especially by addition of manure (Fig. 2D). Earthworm biomass was also higher in the manure treatment compared to all treatments but sewage sludge (Table 3).

### Correlations with soil factors

**Figure 3:**
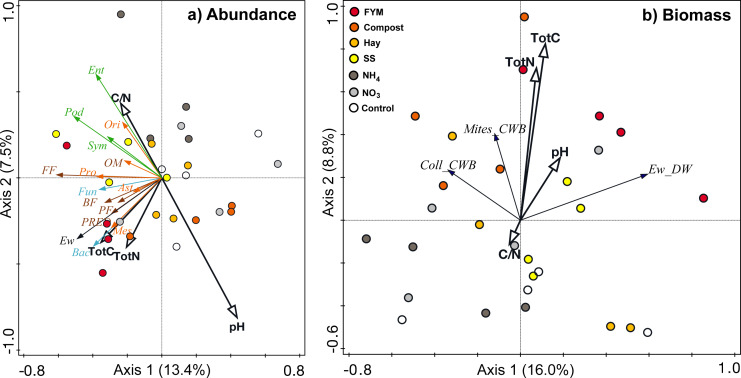
Redundancy analyses correlating (a) abundances and (b) biomass of different soil organism groups to soil chemical factors (total carbon, Tot C; total nitrogen, Tot N; C/N ratio; pH). Microorganisms (light blue)–Fun, fungi, Bac, bacteria; Nematodes (brown)–PF, plant feeders, FF, fungal feeders, BF, bacterial feeders, OM, omnivores, PRED, predators; Springtails (green)–Pod, Podumorpha, Ent, Entomobryomorpha, Sym, Symphypleona; Mites (orange)–Ori, Oribatida, Ast, Astigmata, Pro, Prostigmata, Mes, Mesostigmata; Ew, earthworms (black); CWB, community weighted mean biomass, DW, dry weight, Coll, Collembola (springtails).

Generally, the measured soil factors only explained a minor portion of the variation in the overall abundances (20.9%) and biomasses (24.8%) of the soil organism groups (Fig. 3). Regarding abundances, bacteria, mesostigmatic mites and earthworms correlated positively with the amount of carbon and nitrogen in the soil, while Entomobryomorpha and oribatid mites correlated positively with soil C/N (Fig. 3A). The abundances of the Podumorpha and Entomobryomorpha correlated negatively with soil pH (Fig. 3A). None of the biomasses was clearly correlated to the measured soil factors (Fig. 3B). There were also few correlations with the C/N of the organic amendments; the fungal abundance correlated positively while total springtail abundance and the abundance of Podumorpha correlated negatively (Table 4).

**Table 4:** Spearman rank correlation coefficients between soil biota abundance/biomass and the C/N ratio of the organic amendments. Significant correlations (*p* < 0.05) are indicated in bold.

Soil biota group	C/N amendment
*Microorganisms*	
Fungi (copies/g DW soil)	**0.59**
Bacteria (copies/g DW soil)	0.45
*Nematodes*	
Total abundance (no./g DW soil)	−0.11
Plant feeders	−0.42
Fungal feeders	−0.17
Bacterial feeders	−0.16
Omnivores	−0.42
Predators	−0.09
*Springtails*	
Total abundance (ind./m^2^)	**−0.55**
Podumorpha	**−0.61**
Entomobryomorpha	−0.09
Symphypleona	−0.32
CWB^1^ (µg/ind.)	−0.28
*Mites*	
Total abundance (ind./m^2^)	0.39
Oribatida	−0.04
Astigmata	0.46
Prostigmata	0.18
Mesostigmata	0.26
CWB^1^ (µg/ind)	0.06
*Earthworms*	
Abundance (ind./m^2^)	0.02
Biomass (g DW/m^2^)	0.00

**Notes.**

1 CWB, community weighted mean biomass.

## Discussion

As hypothesized, long-term addition of both mineral and organic fertilizers enhanced the abundances of most groups of soil organisms compared with unfertilized soil, but contrary to our second hypothesis, the mineral fertilizers had an equally stimulating effect on soil biota as organic fertilizers. The lack of major differences in the abundances of various soil community members between organic and mineral fertilizer treatments could be due to positive priming in the plots treated with mineral fertilizers. Mineral fertilization is known to increase the turnover of soil organic matter shortly after application, thus providing easily available nutrients that can improve conditions for a variety of soil biota (Kuzyakov, Friedel & Stahr, 2000). In the long term, mineral fertilizers generally increase the microbial biomass in cropping systems (Geisseler & Scow, 2014), and this effect can propagate in the soil food web and increase soil fauna and erase differences between fertilization treatments. In addition, the increased crop production (i.e., plant dry weight) in the plots with mineral fertilization compared with the other treatments (Kätterer, Börjesson & Kirchmann, 2014) also adds labile C to the soil via root exudates and decaying crop roots, which would positively influence soil biota. The fact that the mineral fertilizer treatments were also supplied with P and K, while the organic amendments were not, could potentially create nutrient imbalances in the soil, although previous studies have shown that systems with organic fertilizers have higher levels of these nutrients than conventional systems (e.g., Clark et al., 1998). Unfortunately, we do not have data on soil nutrient levels, other than nitrogen, and future studies evaluating effects of organic amendments should address effects of possible nutrient imbalances.

In opposition to our third hypothesis, the C/N of the organic amendment did not influence the soil biota, as the more N-rich organic fertilizers had effects similar to those with higher C/N. Nevertheless, fertilization affected the organisms in different ways depending on the type of fertilizer and taxa considered. Both fungi and bacteria were affected by the fertilizers although the ratio between them was not, which is in line with previous findings (Bittman, Forge & Kowalenko, 2005; Kätterer, Börjesson & Kirchmann, 2014). Bacteria and fungi are both taxonomically and functionally extremely diverse groups of organisms. Therefore, there were probably context-dependent responses by the different bacterial and fungal species and functional guilds to the different fertilization treatments that were not captured by only looking at the total community size. This could explain why reported responses in terms of microbial abundances and biomass to increasing soil C after addition of organic matter have been inconsistent (i.e., Witter, Mårtensson & Garcia, 1993; Houot & Chaussod, 1995; Leita et al., 1999; Enwall et al., 2007; Hallin et al., 2009; Cederlund et al., 2014; Kätterer, Börjesson & Kirchmann, 2014). For example, arbuscular mycorrhizal fungal abundance has been shown to be promoted by manure addition (Liu et al., 2020; Ozlu et al., 2019). In addition, microbial performance depends on multiple soil properties, e.g., pH and bulk density, that are also affected by organic amendments (Bünemann, Schwenke & Van Zwieten, 2006). Organic matter can also be more or less readily accessible to microorganisms as it is degraded. Thus, the increased abundance of bacteria in the hay treatment two years after amendment may be due to the recalcitrant nature of hay; this suggestion is supported by long-term results showing that bacterial abundances and biomass only increase in straw or peat amended soils when supplemented with mineral N compared with mineral fertilized soils (Wessén et al., 2010; Cederlund et al., 2014). Overall, our results suggest that in agricultural systems with annual crop rotations and annual tillage, biennial amendments with organic matter promote fungal and bacterial abundances only marginally in the long-term compared with annual addition of mineral fertilizers.

Although there was an overall effect of the fertilizer treatments on the total abundance of nematodes, surprisingly this was not apparent on the nematode feeding group level. In particular, bacterivorous nematodes are known to respond positively to fertilization, especially to organic amendments (e.g., Forge, Bittman & Kowalenko, 2005a; Leroy et al., 2007; Villenave et al., 2010; D’Hose et al., 2018; Grabau, Vetsch & Chen, 2018). Nevertheless, an increase in bacterivore abundance is greatest in the season when the fertilizer is applied (Grabau, Vetsch & Chen, 2018) and in our study, the last application may have been too long ago to see an effect. The lack of responses in bacterial- and fungal-feeding nematodes could also be a reflection of the marginal effects of the treatments on their bacterial and fungal food sources, respectively. For root-feeding nematodes, positive (Weiss & Larink, 1991; Forge, Bittman & Kowalenko, 2005b), neutral (Bulluck, Barker & Ristaino, 2002; D’Hose et al., 2018) and negative effects (Akhtar, 1999; Leroy et al., 2007) of organic fertilizers have been found. However, often only a limited number of fertilizers have been compared in the same study, and in many cases only one type of organic fertilizer was investigated. Altogether, our comprehensive comparison of several organic and inorganic fertilizers indicates that long-term fertilization is a minor factor affecting the occurrence of root-feeding nematodes, which was also suggested by Schmidt et al. (2017). Both long-term mineral (ammonium sulphate) and organic (manure and sewage sludge) fertilizers appeared to promote nematodes in agricultural soils compared with no fertilization. Due to the differences between these fertilizers and the fact that no effects were found at the feeding group level, the biotic and abiotic ecological processes underpinning the observed pattern probably differ between the amendment types.

The mesofauna showed contrasting responses to fertilization depending on the taxa, which, in the case of springtails, was related to differences in soil pH among the treatments. Previous studies have shown increasing numbers of springtails with several different organic amendments (Wang et al., 2015; Platen & Glemnitz, 2016; Leroy et al., 2007), but not with sewage sludge (Andrés et al., 2011). Surprisingly, we found the highest springtail abundance in sewage sludge-treated plots, particularly within the sub-family Podumorpha. Possibly, it was the combination of increasing organic matter and low pH caused by the addition of sewage sludge that benefited the Podumorpha. Mites did not benefit from fertilization *per se* as the total abundance did not differ between unfertilized and fertilized plots, but their abundance was generally enhanced by organic fertilizers compared with mineral fertilizers. The response of mites to fertilizers may be complex because of their diversity of feeding habits and habitat preferences within and among the different sub-orders (Schneider et al., 2004). Nevertheless, manure additions supported relatively high abundances of all mite sub-orders, which agrees with previous results (Minor & Norton, 2004; Cao et al., 2011; Gruss, Twardowski & Hurej, 2018). Our results suggest that the mesofauna is only marginally promoted by long-term fertilization, whether with mineral or organic fertilizers. Whether this depends on small direct effects of the fertilizer treatments or indirect effects on their food sources or other soil fauna is not possible to determine from the present study.

Earthworms feed directly on organic matter and are therefore expected to respond positively to the addition of organic amendments, as well as the quality of the amendments. In accordance with our results, addition of manure has been shown to increase abundance and biomass of earthworms (Edwards & Lofty, 1982; Whalen, Parmelee & Edwards, 1998; Sizmur et al., 2017; D’Hose et al., 2018). Adding hay as an organic amendment has been studied far less, but earthworms do feed on both hay and straw (Al-Maliki & Scullion, 2013). However, field observations have indicated that the size of the straw pieces is important and that straw milled into smaller pieces (<3 mm) is more easily ingested (Sizmur et al., 2017). Studies with rice straw have also shown that the moisture level in the soil is important for its decomposition by earthworms (Gorbunova et al., 2020). The hay used in the present study was chopped into 20 cm long pieces, but other factors related to the conditions in the soil probably also contributed to the response of the earthworms to the addition of hay. Earthworms readily feed on sewage sludge and can even be used for vermicomposting it (Sinha et al., 2009). Therefore, it is not surprising that in the present study the abundance of earthworms increased in the plots with added sewage sludge. The same pattern has also been found in previous studies evaluating addition of sewage sludge in the field (Hamilton & Dindal, 1989; Stöven & Schnug, 2009).

Although presented and mostly discussed separately here, soil organisms interact with each other and influence each other’s populations. As the organism groups were not sampled at the same time during the season, we did not investigate co-occurrences and correlations between them. Even when sampled at the same time, however, it is difficult to draw conclusions about the mechanisms behind co-occurrence patterns (Horner-Devine et al., 2007), especially when analyzing the soil biota at low taxonomic resolution. Nevertheless, we believe that our results based on abundances of all major soil organism groups are adequate for the general questions we are addressing related to the long-term effects of fertilizers on soil biota. To also address questions related to soil health, a higher taxonomic resolution would be needed to decipher the responses to management and performance of the detrimental species compared with the beneficial species within the individual soil organism groups. More long-term studies covering a wider range of C/N ratios than in the present study are also needed, as well as evaluation of long-term annual organic amendments.

## Conclusions

Long-term addition of organic and mineral fertilizers had beneficial effects on the abundances of most soil organisms compared with unfertilized soil, but the responses differed between the soil biota groups. That the effect of organic amendments was similar to mineral fertilizers for some of the groups indicates that biennial organic amendments are insufficient by themselves to boost soil biota and annual additions or combining additions with other practices affecting soil quality, such as no or reduced tillage and other crop rotations, are required to deliver this effect. There had probably been positive transient effects of the amendments on some of the more rapidly-responding groups, like bacteria and bacterial-feeding nematodes. The only organism group that showed a clear positive response to the organic amendments was the earthworms, a group that directly feeds on the added material. Overall, the specific type of fertilizer was more important in determining the numerical and compositional responses of soil biota than whether it was mineral or organic in nature. Our results indicate that both chemical properties, such as pH and C and N levels in the soil, as well as biotic interactions between the soil biota groups are responsible for the observed patterns, and these factors are in turn affected by the fertilizer treatments.

We highlight the fact that accurate assessments of the influence of fertilizers on soil biota communities will be achieved only if a wide range of soil organisms and soil chemistry are investigated concurrently.

##  Supplemental Information

10.7717/peerj.11204/supp-1 Supplemental Information 1 Biennial application rates of dry matter (DM), pH, and amount of carbon and nitrogen in the organic amendments in the long-term fertilization experiment in Lanna, Sweden Total carbon (Ctot), organic nitrogen (Norg), inorganic nitrogen (Nmin), and total nitrogen (Ntot).

10.7717/peerj.11204/supp-2 Supplemental Information 2 Abundance of different nematode feeding groups (individuals per g dry soil) in the long-term fertilization experiment in Lanna, Sweden; mean (SE) RF, root feeders, FF, fungal feeders, BF, bacterial feeders, OM, omnivores, PRED, predators. There were no significant treatment effects (lme, Tukey *p* < 0.05, *n* = 4).

10.7717/peerj.11204/supp-3 Supplemental Information 3 Raw data for soil chemical factors and soil biota groups

## Additional Information and Declarations

### Data Availability

The following information was supplied regarding data availability: The properties of the organic amendments is available as Table S1. The processed nematode data is available as Table S2. The raw data concerning soil chemical factors and all soil biota groups is available as Table S3.
