# Supplementary material for: Type of organic fertilizer rather than organic amendment per se increases abundance of soil biota
Source: PeerJ. 2021 May 7;9:e11204. doi: 10.7717/peerj.11204 (PMC8109005; doi:10.7717/peerj.11204)
Supplement: Supplemental Information 1 — Total carbon (Ctot), organic nitrogen (Norg), inorganic nitrogen (Nmin), and total nitrogen (Ntot). [file peerj-09-11204-s001.docx]

| **Organic amendment** | **DM**  **(Mg ha^-1^)** | **pH** | **Ctot**  **(Mg ha^-1^)** | **Norg**  **(kg ha^-1^)** | **Nmin**  **(kg ha^-1^)** | **Ntot (kg ha^-1^)** |
| --- | --- | --- | --- | --- | --- | --- |
| Grass hay | 8.9 | 5.6 | 3.71 | 179 | 2 | 181 |
| Farmyard manure | 10.1 | 8.7 | 4.04 | 188 | 53 | 241 |
| House-hold compost | 17.1 | 7.4 | 4.16 | 394 | 50 | 444 |
| Sewage sludge | 17.1 | 7.9 | 3.91 | 251 | 193 | 444 |
