# Supplementary material for: Type of organic fertilizer rather than organic amendment per se increases abundance of soil biota
Source: PeerJ. 2021 May 7;9:e11204. doi: 10.7717/peerj.11204 (PMC8109005; doi:10.7717/peerj.11204)
Supplement: Supplemental Information 2 — RF, root feeders, FF, fungal feeders, BF, bacterial feeders, OM, omnivores, PRED, predators. There were no significant treatment effects (lme, Tukey p < 0.05, n = 4). [file peerj-09-11204-s002.docx]

**Table S1** Abundance of different nematode feeding groups (individuals per g dry soil) in a long-term fertilization experiment in Lanna (mean (SE)). RF = root feeders, FF = fungal feeders, BF = bacterial feeders, OM = omnivores, PRED = predators. There were no significant treatment effects (lme, Tukey p<0.05, n=4).

|  | **RF** | **FF** | **BF** | **OM** | **PRED** |  |
| --- | --- | --- | --- | --- | --- | --- |
| Unfertilized | 0.55 (0.13) | 5.67 (1.43) | 2.52 (0.68) | 0.30 (0.06) | 0.62 (0.27) |  |
| Ca(NO_3_)_2_ | 0.42 (0.07) | 7.02 (1.82) | 3.42 (0.40) | 0.45 (0.10) | 0.61 (0.19) |  |
| (NH_4_)_2_SO_4_ | 1.04 (0.26) | 9.76 (1.32) | 4.38 (0.91) | 0.54 (0.26) | 0.29 (0.12) |  |
| Grass hay | 0.60 (0.12) | 6.88 (1.69) | 3.86 (1.49) | 0.25 (0.07) | 0.32 (0.12) |  |
| Farmyard manure | 1.10 (0.17) | 10.29 (2.22) | 5.00 (1.69) | 0.42 (0.07) | 0.72 (0.26) |  |
| House-hold compost | 0.95 (0.06) | 6.60 (0.86) | 2.57 (0.96) | 0.25 (0.02) | 0.42 (0.12) |  |
| Sewage sludge | 1.59 (0.59) | 9.75 (1.61) | 5.40 (1.51) | 0.93 (0.26) | 0.64 (0.30) |  |
